# Metformin inhibits metastatic breast cancer progression and improves chemosensitivity by inducing vessel normalization via PDGF-B downregulation

**DOI:** 10.1186/s13046-019-1211-2

**Published:** 2019-06-04

**Authors:** Ji-Chang Wang, Guang-Yue Li, Bo Wang, Su-Xia Han, Xin Sun, Yi-Na Jiang, Yan-Wei Shen, Can Zhou, Jun Feng, Shao-Ying Lu, Jian-Lin Liu, Mao-De Wang, Pei-Jun Liu

**Affiliations:** 1 Department of Vascular Surgery, First Affiliated Hospital of Xi’an Jiaotong University, Xi’an, 710061 Shaanxi Province China; 2 Center for Translational Medicine, First Affiliated Hospital of Xi’an Jiaotong University, No. 277 of the Western Yanta Road, Xi’an, 710061 Shaanxi Province China; 3 Department of Science and Technology, First Affiliated Hospital of Xi’an Jiaotong University, Xi’an, 710061 Shaanxi Province China; 4 Department of Oncological Radiotherapy, First Affiliated Hospital of Xi’an Jiaotong University, Xi’an, 710061 Shaanxi Province China; 5 Department of Thoracic Surgery, First Affiliated Hospital of Xi’an Jiaotong University, Xi’an, 710061 Shaanxi Province China; 6 Department of Pathology, First Affiliated Hospital of Xi’an Jiaotong University, Xi’an, 710061 Shaanxi Province China; 7 Department of Medical Oncology, First Affiliated Hospital of Xi’an Jiaotong University, Xi’an, 710061 Shaanxi Province China; 8 Department of Breast Surgery, First Affiliated Hospital of Xi’an Jiaotong University, Xi’an, 710061 Shaanxi Province China; 9 Department of Neurosurgery, First Affiliated Hospital of Xi’an Jiaotong University, No. 277 of the Western Yanta Road, Xi’an, 710061 Shaanxi Province China; 10 Key Laboratory for Tumor Precision Medicine of Shaanxi Province, First Affiliated Hospital of Xi’an Jiaotong University, No. 277 of the Western Yanta Road, Xi’an, 710061 Shaanxi Province China

**Keywords:** Metformin, Metastatic breast cancer, Vessel normalization, Chemosensitization, PDGF-B

## Abstract

**Background:**

Vascular maturity and functionality are closely associated with tumor progression and chemosensitivity. The antidiabetic agent metformin has shown its ability to inhibit tumor angiogenesis in metastatic breast cancer models. However, it remains unclear if or how metformin remodels the abnormal vasculature of metastatic breast cancer, while inhibiting angiogenesis.

**Methods:**

Metastatic breast cancer models were constructed to compare microvessel density (MVD), vascular maturity and function, lung metastasis and chemosensitivity in metformin-treated or untreated mice. Protein array assay and transcriptome sequencing were performed for genetic screening. Lentiviral shRNA-PDGF-B transfection was used for observing the contribution of PDGF-B knockdown to metformin’s vascular effects.

**Results:**

Metastatic breast cancers were characterized by an excessively angiogenic, immature and morphologically abnormal vasculature. Compared to control, metformin significantly reduced MVD, leakage and hypoxia, and increased vascular mural cells coverage and perfusion, namely, “vessel normalization”. Metformin at human blood concentrations had no direct effect on the migration and proliferation of cancer cells. Based on that, reduced lung metastasis of the primary tumor and improved chemosensitization by metformin were assumed to be mediated via metformin’s vascular effects. Further results of genetic screening and in vivo experiments showed that the downregulation of platelet-derived growth factor B (PDGF-B) greatly contributed to the metformin-induced vessel normalization.

**Conclusions:**

These findings provide pre-clinical evidences for the vascular mechanism of metformin-induced metastasis inhibition and the chemosensitization of metastatic breast cancers.

**Electronic supplementary material:**

The online version of this article (10.1186/s13046-019-1211-2) contains supplementary material, which is available to authorized users.

## Background

Angiogenesis that mediates the formation of new blood vessels serves as a hallmark for cancers [1], of which the critical role in cancer progression has now been widely accepted [2]. Hence, anti-angiogenic drugs (AADs) have been extensively developed whose usage constitutes a major modality of anti-tumor therapy [3]. However, mechanisms underlying AADs-induced antitumor effects remained unclear. Currently, there are two major hypotheses highly relevant to AADs-related antitumor activities. One offers a possible mechanism that cancer cells are killed through the blocking of blood supply by AADs via the inhibition of tumor angiogenesis [2]. Up until now, this hypothesized tumor-starving mechanism has not been clinically verified. Another hypothesis involves the remodeling of the remaining abnormal vessels [4, 5], also known as “vessel normalization”. In the latter hypothesis, the drugs not only suppress both the growth and metastasis of the tumor but also enhance the chemosensitization of cancer cells by improving the vascular maturity and functionality, and ameliorating tumor hypoxia [6].

Conventional therapies targeting tumor angiogenesis is efficacious (in terms of survival benefit) only for some cancers, such as colorectal cancer and renal cell carcinoma, etc., but not for others (e.g. breast cancer, melanoma) [6, 7]. Anti-angiogenic benefit in term of survival cannot be seen in all patients with cancers [8, 9], which have been clinically demonstrated to be responsive to anti-angiogenic therapies. For instance, bevacizumab added to chemo-drug did not significantly improve the overall survival of the patients with metastatic breast cancers [9]. This is partially due to the lack of vascular parameters available for predicting the treatment efficacy [10]. Moreover, intrinsic and acquired resistance have been shown to even impair the survival benefit already achieved clinically in some cancer patients [3, 11]. Thus, there is a pressing need for researchers to develop a more effective treatment regimen.

Population- and clinic-based studies have demonstrated the potential anti-proliferative and anti-metastatic activities of the antidiabetic agent metformin, a member of biguanides, when used in cases with malignant diseases [12–14]. Data from preclinical studies have revealed the pleiotropic effects of metformin [15, 16]. However, the mechanisms of metformin’s effects in carcinogenesis were not fully understood, and more details concerning metformin’s effects should be further studied. The anti-angiogenesis potential of metformin has recently been reported by several laboratories [17–19]. However, little is known to date about if or how metformin remodels the abnormal tumor vasculature, while inhibiting angiogenesis. Since vascular maturity and functionality are closely associated with hypoxia and metastasis [20], further researches with a focus on the vascular mechanism would be hugely meaningful. Additionally, biguanides also have the potential to enhance the in vivo toxicity of chemo-drug for cancer treatment [21, 22], but it was still unclear whether this chemosensitization involves a vascular mechanism.

The aim of the present study was to investigate the effects of metformin on vascular maturity and functionality and angiogenesis. Further results of genetic screening imply the deep involvement of platelet-derived growth factor B (PDGF-B) in metformin-induced vessel normalization.

## Methods

### Cell culture, proliferation, colony formation and migration assays

HUVECs and murine 4T1 and human MDA-MB-231 metastatic breast cancer cell lines were obtained from the American Type Culture Collection (ATCC, Manassas, VA) and cultured in Dulbecco’s Modified Eagle’s medium (DMEM) (Invitrogen) supplemented with 10% fetal bovine serum (FBS). All cell lines used in the study were not listed in the database of commonly misidentified cell lines maintained by International Cell Line Authentication Committee (ICLAC). Cell line Cross-Contamination was tested using the Short Tandem Repeat (STR) genotyping analysis method. Mycoplasma contamination was tested using Myco-Test Kit of MP Biomedicals (No.093030000) every three months, or when the growth rate and morphology of the cell lines were found to be abnormal. All cell lines were cultured and maintained in an atmosphere consisting of CO^2^ (5%) and the room air (95%) at 37 °C. In vitro migration was analyzed by inoculating cancer cells in the upper chamber of a transwell (Millipore; 8 μm pore insert). To assess in vitro colonization ability, cancer cells were cultured in a 6-well culture plate, which were finally fixed and stained with crystal violet solution (C0775, Sigma). Cellular proliferation rate was measured by counting the number of cells in the culture dish (6 cm diameter) each day.

### Chemicals and reagents

Metformin (No.13118), cyclophosphamide (CTX, No.13849), cisplatin (CPT, No.13119) and imatinib (No.13139) were purchased from Cayman Chemical. The active 4-hydroxy-CTX (4-OH-CTX) was obtained by the ozonization of cyclophosphamide using the method described previously [23, 24].

### Protein array assay

To analyze the proteome profile of angiogenesis-related factors, mouse angiogenesis array (ARY015, R&D System) was incubated with fresh cellular lysates containing 1 mg protein according to the manufacturer’s instructions. The RapidStep™ ECL reagent (No.345818, Millipore) was used, and spot intensities were measured using the NIH ImageJ software (Bethesda, MD) for data capture.

### Mouse models

All animals were obtained from Animal Experiment Center of Xi’an Jiaotong University. To assess the in vivo tumor growth, 5Х10^5^ 4T1 and 2Х10^6^ MDA-MB-231 cells were suspended in 100 μL precoated PBS, and then injected into the fat pad of the 4th left mammary gland of mice (4T1: female BALB/c; MDA-MB-231: female nude mice). After 6–8 weeks, the mice without obvious abnormality in appearance were randomly divided into different groups (8 mice per group) when the mean tumor volume reached about 50–100 mm^2^. For the observation of chemosensitization, tumor-bearing mice were pretreated with metformin (orally) for 5 days before receiving the intraperitoneal injection of CTX. Before the experiment started formally, the proper sample size that ensures adequate power for a statistical difference was estimated using the following formula: *N* = 2·[(u_0.05)_ + u_(0.10)_)·S/X]^2^, “S” indicates standard deviation of the overall sample, and “X” indicates the difference of the mean tumor weight between two groups. Tumor volume was measured with a caliper every two or three days and calculated using the formula V = 0.523•[a^2^•A] (“a” indicates the minor tumor axis; “A” indicates the major tumor axis). To observe the change of lung metastasis (4 T1) from primary tumors, tumor-bearing mice were fed for at least 28 days after inoculation.

### Flow cytometry analysis

Necrotic and late apoptotic cells were labeled with propidium iodide (PI) at a concentration of 5 μg/mL, and PI^+^ dead cells were identified by Flow Cytometry (BD Bioscience, USA).

### PDGF-B knockdown by shRNA

Lentivirus-mediated PDGF-B silencing was performed by transfecting 4T1 cells with control shRNA (against scrambled sequence) or mouse PDGF-B shRNA. The transfection procedure was carried out according to the manufacture’s protocol. Positively transfected cells were selected using puromycin, and the silencing efficiency was investigated with the quantitative real-time polymerase chain reaction (PCR). The primers for detecting mRNA level of mouse PDGF-B (NM_011057.3 → NP_035187.2, CCDS: CCDS27656.1) were as follows: the forward primer: 5`-TCTCTGCTGCTACCTGCGTCT-3`, the reverse primer: 5`- CAGCCCCATCTTCATCTACGG -3`.

### Transcriptome sequencing assay

100 mg tissue samples from the 4T1 tumors in metformin-treated or untreated mice were extracted quickly and then put on the ice, and each sample was immediately cryopreserved with liquid nitrogen. Messenger RNA (mRNA) extraction, cDNA synthesis, PCR enrichment, library construction, quality control and sequencing were performed by Beijing Biomarker Corporation (China, Beijing). 3 independent samples in each group were used for gene expression analysis. Heatmaps were presented to show the change of gene expression levels using Prism 7.0 (GraphPad, USA).

### Immunofluorescence, Histomorphometry and H&E Staining

Mouse tissues were fixed in 4% PFA for 12 h at 4 °C, and sequentially dehydrated in the 20 and 30% sucrose solutions, respectively. For the 2D and 3D confocal imaging, tissue samples were cut into 6 μm-thick and 40 μm-thick sections, respectively. The prepared sections were stored in a − 80 °C Laboratory Freezer (DW-86L728J, Haier). Single or double immunostaining was performed with the following antibodies. Primary antibodies: CD31 (anti-rabbit, ab28364, Abcam; anti-Rat, ab7388, Abcam), PDGF-B (BA0519–2, Boster), α-SMA (BM0002, Boster), VE-cadherin (No.138101, BioLegend), cl-PARP (#9542, Cell Signaling), PCNA (BM3888, Boster), cl-Caspase-9 (#9542, Cell Signaling), cl-Caspase-3 (#9661, Cell Signaling); NG-2 (R&D, MAB6689). Secondary antibodies: Alexa fluor 488-conjugated Goat anti-Rabbit antibody (A-11008, Invitrogen), Alexa fluor 488-conjugated Goat anti-Rat antibody (A-11006, Invitrogen), DyLight 550-conjugated Donkey anti-Rat antibody (SA5–10027, Invitrogen), Alexa fluor 546-conjugated Goat anti-Rabbit antibody (A-11010, Invitrogen), Alexa fluor 546-conjugated Donkey anti-Mouse antibody (A-10036, Invitrogen), Alexa fluor 647-conjugated Donkey anti-Mouse antibody (A-31571, Invitrogen), Alexa fluor 546-conjugated Goat anti-Rat antibody (A-11081, Invitrogen). Sections were washed with 0.1% PBST, blocked with 5% BSA in PBST at 37 °C for 1 h, and permeabilized with the 0.2% triton X-100 solution for 15–30 min.

For fluorescent 3D-reconstruction, 40 μm-thick sections were treated with the 0.1% trypsin retrieval solution at 37 °C for 15–20 min to get enhanced signal for signal detection. Sections were then incubated with primary antibodies diluted in 5% BSA (0.1% PBST) at 4 °C for no less than 24 h, followed by staining with the appropriate, fluorescently conjugated secondary antibodies. Nuclei were counter-stained by DAPI (2-5 μg/mL) at the room temperature for 15 min before the fluorescent imaging. The fluorescent single- or multi-layer images (2.5–3.5 μm per layer) were obtained using confocal laser scanning microscopy (Leica, German). Software of LAS AF Lite (Leica, German) was used to perform the 3D-reconstrution of CD31 or CD31/α-SMA fluorescent signaling. Furthermore, 5–10 fields (20 x magnification) per tumor were randomly selected and analyzed [25].

To observe the perfusion status, perfused vessels were labeled by the intravenous injection of 20 mg/kg Rhodamine-labeled lectin (RL-1102, Vector Labs) 15 min before the intracardiac perfusion of 40 ml 4% paraformaldehyde with a flux of 10 ml/min. Tumors were then extracted, fixed in 4% PFA for 1 h at 4 °C, sequentially dehydrated, embedded in OCT (Tissue-Tek #4583, Sakura Finetek, USA), and cut into 6 μm-thick sections. All perfused or un-perfused vessels were immunostained with anti-CD31 antibodies (ab28364, Abcam) followed by staining with Alexa fluor 488-conjugated Goat anti-Rabbit secondary antibodies (A-11008, Invitrogen). For the observation of the vascular leakage of tumors, 100 mg/kg Fluorescein Isothiocyanate (FITC)-conjugated Dextran (70kD, No.53471, Sigma) was intravenously injected into tumor-bearing mice 10 min before tumors were harvested. To detect tumor hypoxia, 60 mg/kg PIMO (Hypoxyprobe Inc.) was intravenously injected into the tail vein of tumor-bearing mice 90 min before tumors were wholly extracted. PIMO^+^ hypoxic cells in tumor sections were then immunostained with anti-PIMO antibody (Hypoxyprobe Inc.) according to the manufacturer’s instructions.

The H&E-stained paraffin sections (4 μm) of those fixed tumors or lung tissues were assessed for tumor necrosis, hemorrhage and metastatic nodules. To observe the chemosensitization, tumor sections from Cisplatin-treated mice were stained with anti-Cisplatin-modified DNA antibody (GTX17412, Genetex). Paraffin-embedded sections were sequentially incubated with secondary antibodies (SV0002, Boster), 3, 3′-diaminobenzidine (DAB, ZLI-9017, ZSGB-Bio) and the hematoxylin (H9627, Sigma) solution.

### Statistical analysis

Quantitative analysis was performed using the Prism 5.0 or 7.0 software (GraphPad, San Diego, CA). All quantitative data were represented by mean ± SEM. Kolmogorov-Smirnov normality test was performed to analyze the normal distribution, and coefficient of variation (CV) was used to estimate the variation of data within each group. When CV was greater than 15%, the data was considered to be abnormal. Bartlett’s test was performed for investigating the homogeneity of variances between the groups. For any set of data which was not normally distributed, nonparametric Wilcoxon or Kruskal-Wallis test was performed to investigate the statistical difference between two or multiple independent samples. The statistical significance between two groups and multiple groups was defined as *P* < 0.05 by two-tailed student’s t test or one-way ANOVA t-test. Two-way ANOVA analysis was performed when an additional factor or variant was involved in the experiment.

## Results

### Metastatic breast cancers were characterized by an excessively angiogenic and immature vasculature

**Fig. 1:**
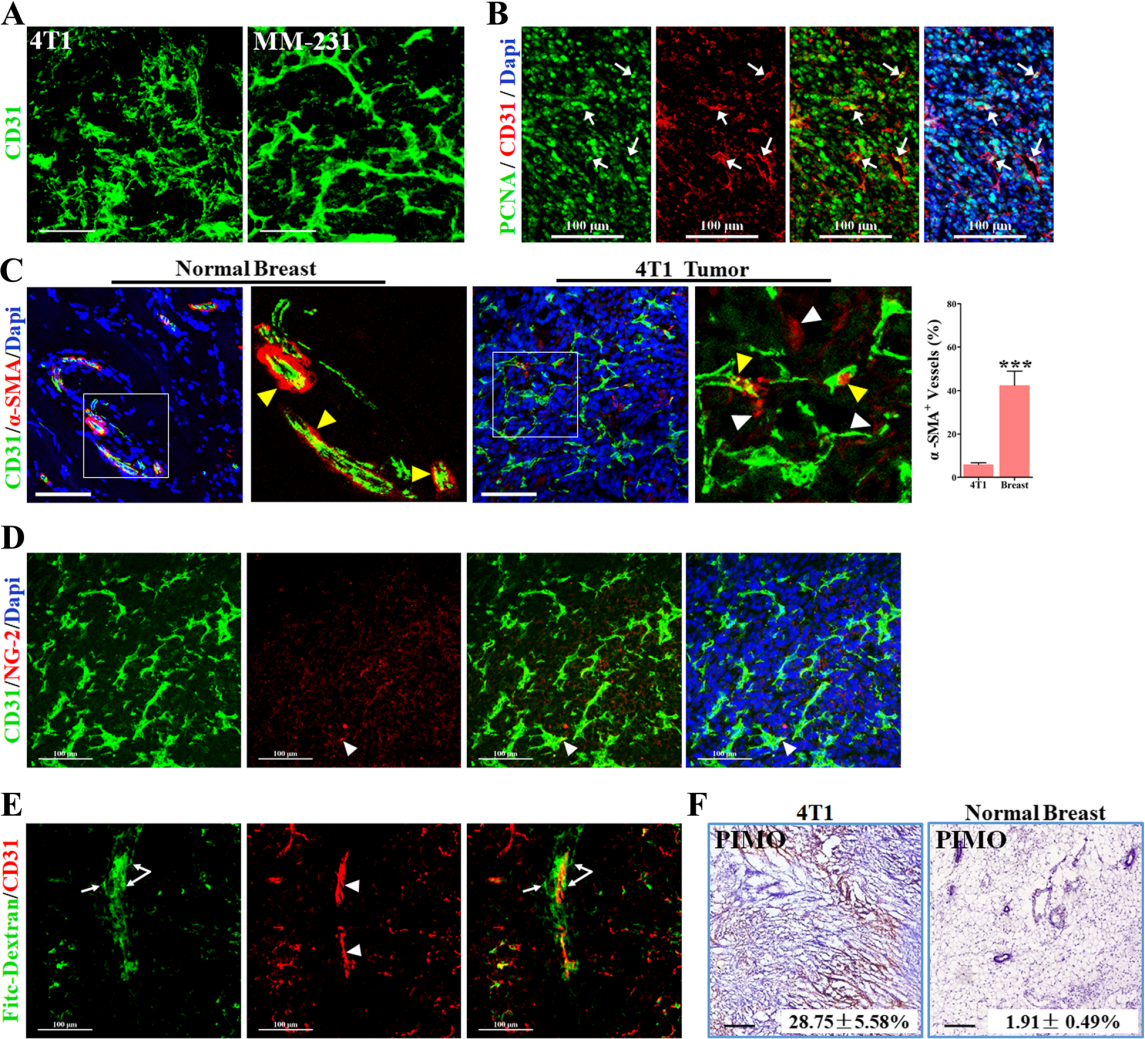
Vascular morphology, maturity and functional status of the metastatic breast cancers. 4 T1 and MDA-MB-231 metastatic breast cancer cells were orthotopically injected into the fat pad of mice. (**a**) 3D-reconstruction of CD31^+^ vessels in 4 T1 and MDA-MB-231 (MM-231) tumors from untreated mice. Tumor sections were stained with an anti-CD31 antibody. Bars: 100 μm. (**b**) Immunofluorescent double-staining for PCNA (Green) and CD31 (Red) in sections of untreated CT-26 tumors. White arrows indicate PCNA nucleus positive endothelial cells that are proliferating. Nuclei were counterstained with DAPI. Bars: 100 μm. (**c**) Double immunostaining (Left) for CD31 (Green) and α-SMA (Red) in frozen sections of normal breast and 4 T1 tumors, and quantification (right) of percentage of α-SMA^+^/CD31^+^ vessels (of CD31^+^ vessels; *n* = 8). White and yellow triangles indicate disassociated vascular smooth muscle cells (VSMCs) and associated VSMCs, respectively. Nuclei were counterstained with DAPI. Bars: 100 μm. (**d**) Double immunostaining for CD31 (Green) and NG-2 (Red) in frozen sections of untreated 4 T1 tumors. White triangle indicates the pericyte associated with the vessel wall. Bars: 100 μm. (**e**) Representative images showing FITC-conjugated dextran perfused CD31^+^ vessels (Red) in 4 T1 tumors from untreated mice. FITC-conjugated dextran was injected through the tail vein in advance. White triangles indicate CD31^+^ vascular lumen containing FITC-Dextran; white arrows indicate dextran leaking outside the vessel wall. Bars: 100 μm. (**f**) Representative image for showing Pimonidazole (PIMO; brown) staining, and quantification of PIMO^+^ hypoxic areas in 4 T1 tumors and normal breast of BALB/c mice (Lower Right Corner, *n* = 8). Bars: 100 μm. Quantitative data are indicated as mean ± SEM. ***p < 0.001

Both MDA-MB-231 and 4T1 breast cancer cell lines were characterized by distant lung metastasis when orthotopically implanted, thus being selected. To observe the vascular morphology of both tumors, the 3D reconstruction of those CD31-stained vessels was performed. Microvessels in both cancers manifested the strong abilities of sprouting and branching (Fig. 1a), and were distinctly larger. This kind of vascular phenotype was similar with what was found in angiogenic cancers. It was further confirmed that a large number of vessels were found to be PCNA^+^ in 4T1 cancers (53.4%), indicating that endothelial cells (ECs) were proliferative (Fig. 1b). These data suggested the involvement of excessive angiogenesis in the progression of metastatic breast cancers.

Next, we focused on the vascular maturity and function. Vascular lumens in the normal breast tissue were extensively covered by vascular smooth muscle cells (VSMCs, marker: α-SMA), a common vascular mural cell, presenting an inerratic lumen morphology (Fig. 1c). However, vessels in the 4T1 cancer were poorly covered by VSMCs. Considering that α-SMA was reported to be abundantly expressed in cancer-associated fibroblasts, the pericyte coverage was further examined using a NG-2 antibody. Consistently, only few NG-2^+^ pericytes (PCs) covered the vessel lumen (indicated by white triangles in Fig. 1d). These data suggested that vessels in metastatic breast cancers were structurally immature. It was worth noting that most of VSMCs and PCs were disassociated or detached from the vascular lumen (Fig. 1c and d).

### Metastatic breast cancers were hypoxic with leaky vessels

Hypoxia and the vascular leakage of tumors were further investigated, since the poor vascular maturity contributes to both [26, 27]. Vessel leakage was detected by the intravenous injection of the FITC-conjugated Dextran (70kD) and the counterstaining with CD31. In 4T1 cancer, a large amount of FITC-conjugated dextran was found to be located outside the CD31^+^ vessel (Fig. 1e, white arrows), indicating that it was extravasated from the blood vessel to extravascular regions. To observe the hypoxic status, PIMO, a reagent for detecting hypoxia, was intravenously injected to tumor-bearing mice after 2 weeks of administration. Further IHC staining showed that 28.75 ± 5.58% areas were stained by the hypoxic marker PIMO in 4 T1 tumors (Fig. 1f), while the normal breast tissue was almost devoid of any hypoxic region. As a key regulator of angiogenesis in cancer, hypoxia was then investigated to show if there was a discrepancy in hypoxic areas (%) between hypo- and hyper-vascular regions. As shown in Additional file 1: Figure S1A and S1B, the hypoxic fraction of the hyper-vascular region was significantly higher than that of the hypo-vascular region. These evidences suggest metastatic breast cancer showed signs of leaky vessel and hypoxia, which were closely associated with the immature vasculature.

### The cell non-autonomous mechanism of metformin in inhibiting the growth and metastasis of tumors

**Fig. 2:**
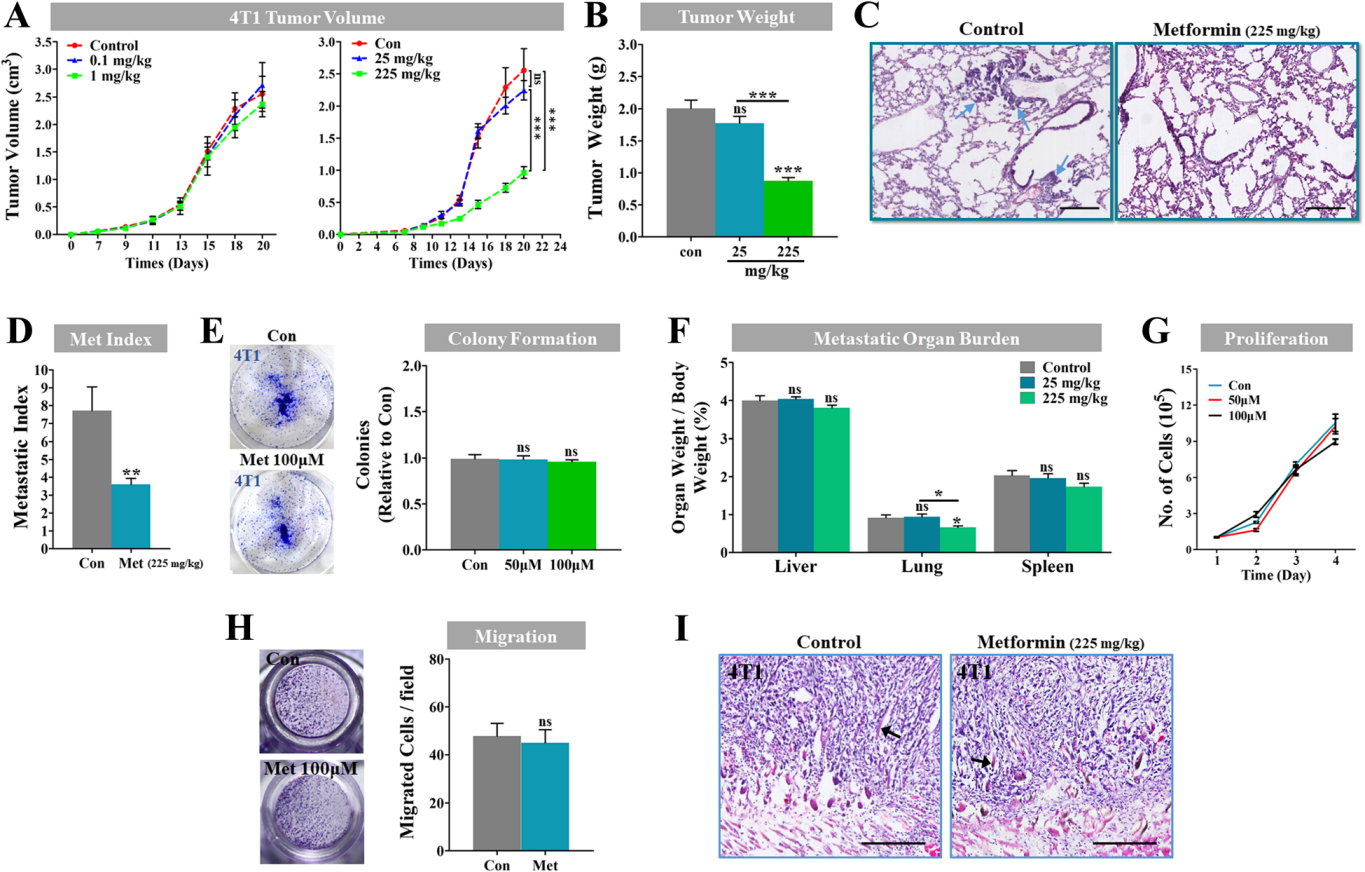
Effects of metformin on the growth and metastasis of the metastatic breast cancers. (**a** and **b**) Growth curve (cm^3^) and measurement of weight (g) of 4 T1 tumors from BALB/c mice untreated or treated with metformin at different concentrations (0.1, 1, 25 and 225 mg/kg•day; *n* = 8). (**c** and **d**) H&E staining for 4 T1 primary tumor lung metastasis, and quantification of 4 T1 metastatic index (metastatic nodules per gram of the primary tumor; n = 8). Blue arrows indicate metastatic nodules in lung. (**e**) Representative images of in vitro colonization of 4 T1 cancer cells, which were untreated or persistently treated with 50 μM or 100 μM metformin (repeated for 5 times). Cancer cells were inoculated into each well of a 6-well culture plate. (**f**) Bar graphs showing reduced lung weight, and comparable weights of liver and spleen of mice untreated or treated with metformin at concentration of 225 mg/kg•day or 25 mg/kg•day (*n* = 6). Organ weight was normalized for body weight. (**g**) In vitro proliferation curve of 4 T1 cancer cells untreated or treated with metformin at concentrations of 50 or 100 μM (repeated for 5 times). (**h**) Representative images showing 4 T1 cancer cell migration in vitro, and quantification of migrated cancer cells per 400 X field (*n* = 5). (I) H&E staining of paraffin-processed tumor tissues from metformin-treated or untreated mice, showing the comparably direct invasion of cancer cells to the peritumoral muscle. Black arrows indicate muscle fibers penetrating into the tumor. Bars: 200 μm. Quantitative data are indicated as mean ± SEM. **p < 0.01; ***p < 0.001

To bring out the clinical antitumor effect of metformin, its routine clinical dose (about 30 mg/kg•day) widely prescribed in antidiabetic use was increased to 225 mg/kg•day for anti-tumor use in the mice by the body surface area normalization [28]. To exclude any low dose-associated hermetic response to metformin, low dose groups (0.1, 1 and 25 mg/kg•day) were concurrently set up. The dose of 225 mg/kg•day was referred to as the clinically relevant dose of metformin. As shown in Fig. 2a, 4T1 cancer did not respond to low doses of metformin (0.1, 1 and 25 mg/kg•day), indicating the absence of any hermetic dose response for taking metformin. Compared to the control, metformin at a dose of 225 mg/kg•day significantly reduced the growth of 4T1 cancers by 63.7% (Fig. 2a and b).

Furthermore, the clinically relevant dose of metformin also greatly decreased the lung metastasis index by 53.9% (Fig. 2c and d). As the metastasis index indicates the number of metastatic nodules per gram of the primary tumor, the inhibition of tumor metastasis may be independent of the direct suppression of the primary tumor growth. Intriguingly, however, metformin did not alter the in vitro colonization of 4T1 cells (Fig. 2e), while inducing great suppression of the metastatic burden of the lungs (Fig. 2f), but not of the liver or the spleen.

Then, it was explored whether metformin directly affected the proliferation and the metastasis of breast cancer cells. Since the plasma concentration of metformin was estimated to be approximately 60 μM in patients, both 50 μM and 100 μM concentrations were used for further in vitro analyses. At both doses, metformin did not alter the proliferation and the migration of breast cancer cells in vitro (Fig. 2g and h). In addition, metformin-treated 4 T1 cancers did not have the same sharp, demarcated borders as the control (Fig. 2i), indicating that metformin had no effect on the direct invasion of cancer cells to the peritumoral muscle. Overall, these data suggested that the antimetastatic and antiproliferative effects of metformin probably were not mediated by a cancerous cell-autonomous mechanism.

### Metformin enhanced the susceptibility of breast cancer cells to chemotherapy

**Fig. 3:**
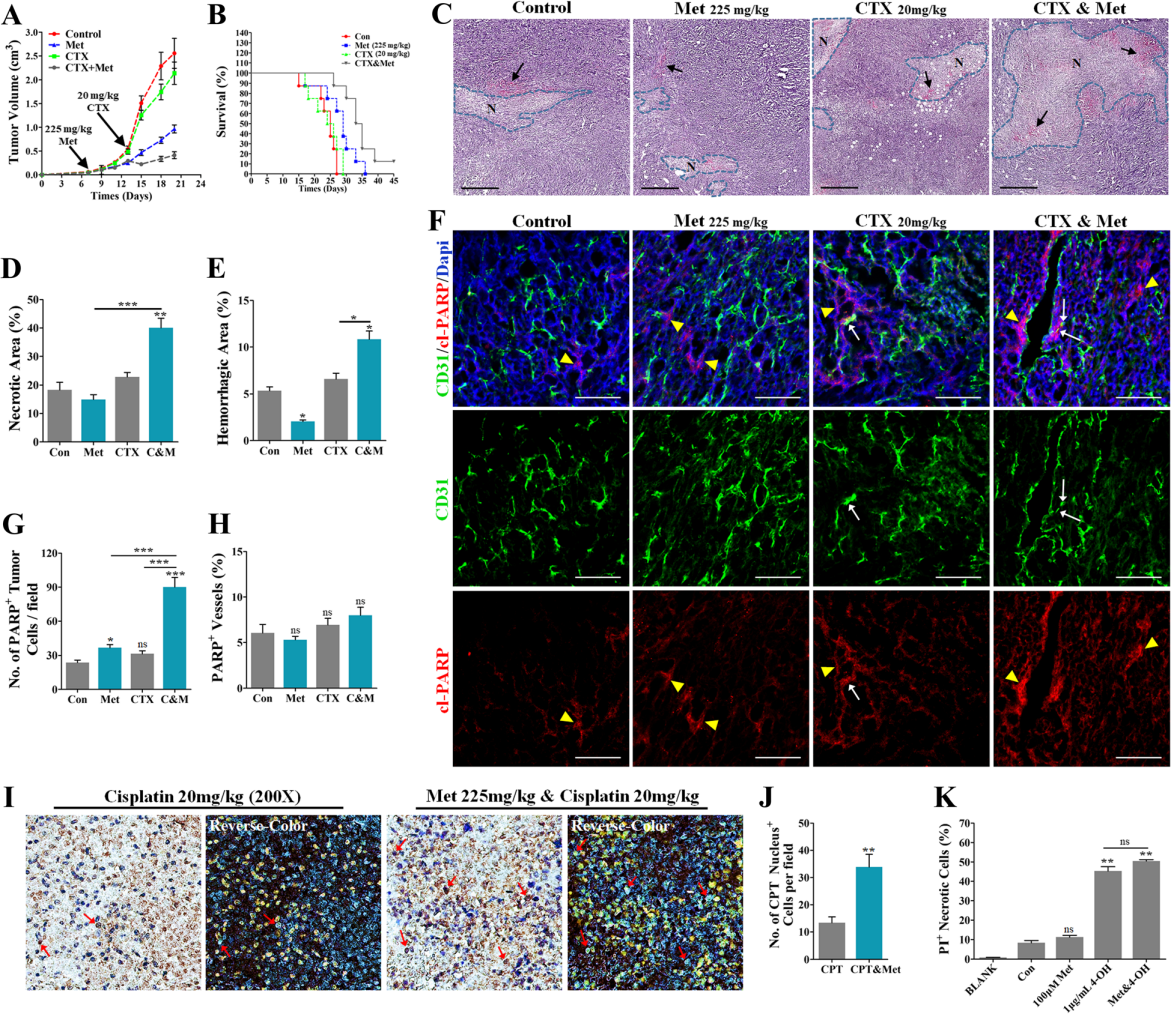
The in vivo effects of metformin pretreatment on cancer responses to cyclophosphamide (CTX). (**a** and **b**) Growth curve and survival curve of 4 T1 cancers from mice untreated or treated with metformin (225 mg/kg•day), low dose CTX (Cyclophosphamide, 20 mg/kg•day) or the combined treatment (*n* = 8). Mice were pretreated with metformin for 5 days before receiving CTX. (C-E) (**c**) H&E staining of sections of 4 T1 cancers, and quantification of (**d**) necrotic and (**e**) hemorrhagic areas, revealing enhanced cancer responses to CTX by metformin pretreatment (n = 8). “N” indicates “Necrosis”; black arrows indicate hemorrhage; “C & M” indicates the combination of CTX and metformin. Bars: 200 μm. (**f**) Double staining for CD31 and PARP of 4 T1 tumor sections, and quantification of (**g**) PARP^+^ CD31^+^ cancer cells and (**h**) PARP^+^ CD31^+^ vessels (n = 8). Tumor-bearing mice were treated with metformin (pretreatment for 5 days), CTX or combined treatment. White arrows indicate PARP^+^ endothelial cells (ECs); yellow triangles indicate PARP^+^ cancer cells. (**i**) Representative images showed increased cisplatin-DNA adducts in 4 T1 tumors from metformin pre-treated mice (225 mg/kg•day, lasting for 5 days). After that, mice were treated with intraperitoneal injections of cisplatin (CPT, 5 mg/kg, every 2 days). The right column refers to the reverse-color image of the left column. Red arrows indicate the cells with positive cisplatin-DNA adducts. Magnification:200Х. (**j**) Quantification of cells with nuclear cisplatin adduct in 4 T1 cancers (*n* = 8). (**k**) Quantification of PI^+^ necrotic ECs and 4 T1 cells in vitro upon combined 4-OH (activated CTX) treatment (n = 5). Quantitative data are indicated as mean ± SEM. *p < 0.05; **p < 0.01; ***p < 0.001

Vascular maturity and functionality have been reported to be associated with chemosensitization. Therefore, the effects of the metformin pretreatment on in vivo sensitivity of cancer cells to low-dose cyclophosphamide (CTX, 20 mg/kg•day) was investigated. Tumor-bearing mice were orally pretreated with metformin starting from the seventh day after inoculation. Compared to the single CTX treatment, the pretreatment combining of CTX and metformin resulted in a more significant anticancer effect and greatly prolonged the survival time of the tumor-bearing mice (Fig. 3a and b). This metformin-mediated chemosensitization was accompanied by the aggravation of tumor necrosis and hemorrhage (Fig. 3c-e).

Double staining for CD31 and cl-PARP further verified the phenomenon described above. Compared to single CTX group, the combined metformin pretreatment resulted in a significant increase of the number of cl-PARP^+^ 4T1 cells (Fig. 3f-h). Interestingly, most of these apoptotic cancer cells were located close to CD31^+^ vessels, suggesting the involvement of the vascular mechanism in the enhancement of the toxicity of chemo-drug. It is worth noting that the combined metformin pretreatment did not increase the proportion of PARP^+^ vessels in 4T1 cancer (Fig. 3h). Furthermore, metformin pretreatment significantly increased the number of cisplatin-DNA adduct-positive 4T1 tumor cells (Red arrows; Fig. 3i and j), suggesting that more cytotoxic drugs were delivered to the tumor. More importantly, metformin did not have the potential to enhance CTX-induced cell necrosis (indicated by propidium iodide (PI)^+^ cells, Fig. 3k) in vitro.

### Metformin inhibited angiogenesis and improved vascular maturity

**Fig. 4:**
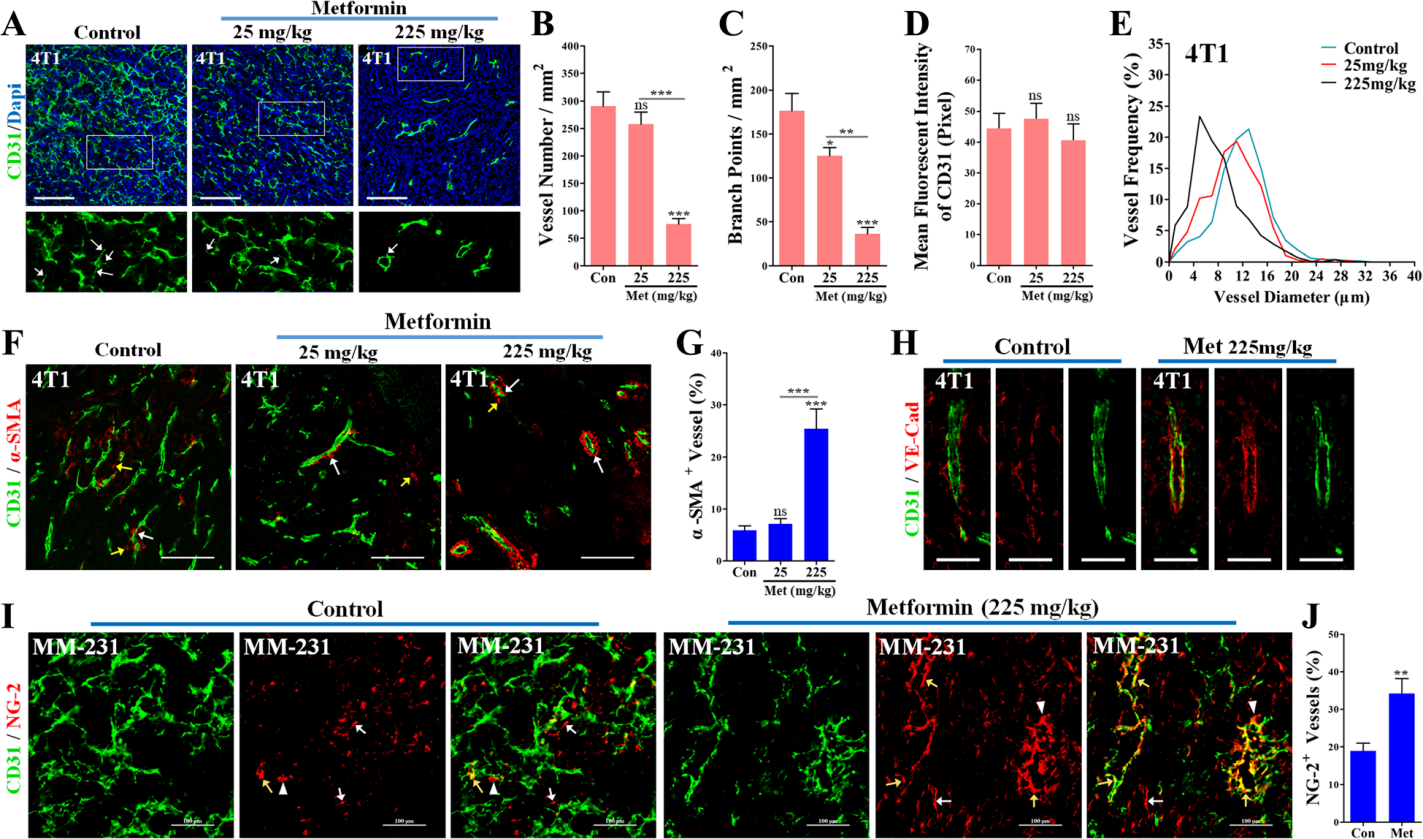
Effects of the metformin administration on angiogenesis and the vascular maturity. (**a**) CD31 immunostaining in sections of 4T1 tumors from mice untreated or treated with metformin at concentrations of 25 mg/kg•day or 225 mg/kg•day. White arrows indicate vascular sprouts of microvessels. Bars: 100 μm. (B and C) quantifications of vascular (**b**) sprouts and (**c**) branches (per mm^2^; *n* = 8). (**d**) Quantification of mean fluorescent intensity of CD31 signaling of vessels in 4 T1 tumors (n = 8), revealing the unaffected CD31 expression level of vessels. (**e**) Analyzes of diameter distribution of vessels in 4T1 tumors from untreated or metformin-treated mice (*n* = 8). (**f**) Double immunostaining for CD31 (Green) and α-SMA (Red) in frozen sections of 4T1 tumors from untreated or metformin-treated mice, and (**g**) quantification of percentage of α-SMA^+^ CD31^+^ vessels (of CD31^+^ vessels; n = 8). White arrows indicate vessels with VSMCs coverage; yellows arrows indicate VSMCs disassociated with vessels. Bars: 100 μm. (**h**) Double immunostaining for CD31 and VE-cadherin of 4T1 tumor sections, revealing continuous and more abundant in metformin-treated than untreated tumors. Bars: 50 μm. (**i**) Double staining for CD31 (green) and NG-2 (red), revealing more vessels with pericyte coverage in metformin-treated than untreated MDA-MB-231 (MM-231) tumors. White and yellow arrows indicate pericytes disassociated and associated with vessels, respectively; white triangles indicate pericytes that are partially associated with vessels. Bars: 100 μm. (**j**) Quantification of NG-2^+^ CD31^+^ vessels (of CD31^+^ vessels) in MDA-MB-231 tumors (*n* = 8). Quantitative data are indicated as mean ± SEM. *p < 0.05; **p < 0.01; ***p < 0.001

Angiogenesis inhibition is one of the mechanisms suppressing the tumor growth [29, 30]. Therefore, it was then investigated whether metformin affects breast cancer angiogenesis. CD31 staining for microvessels showed that normal dose of metformin (225 mg/kg) significantly reduced the micro-vessel density (MVD) and vascular branching points (Fig. 4a-c). The sprouting ability of the micro-vessels in metformin-treated 4T1 tumors were weaker than in the control group (Fig. 4a), while the CD31 signal intensity did not differ between groups (Fig. 4d). In addition, metformin induced a shift of the size of the tumor vessels towards a smaller one (Fig. 4e).

The inhibition of angiogenesis can often be accompanied by a change of vascular maturity [31, 32]. VSMCs and pericytes coverage, the status of the vascular basement membrane and the signatures of vascular maturity [4] were observed by the staining of α-SMA, NG-2 and VE-Cadherin. Metformin-treated 4T1 tumors exhibited a higher percentage of vessels with α-SMA^+^ VSMCs coverage (White arrows, Fig. 4f and g). It is worth noting that metformin-treated 4T1 tumors had fewer VSMCs disassociated with vessels than those of the control (Yellow arrows, Fig. 4f). In addition, less VSMCs disassociated with vessels were found in 4T1 tumors of the metformin-treated mice. Metformin administration shifted the discontinuous VE-Cadherin^+^ vascular basement membrane towards a continuous phenotype while increasing its abundance (Fig. 4h). Consistently, metformin treatment resulted in a significant increase of the percentage of vessels with the coverage of NG-2^+^ pericytes in orthotopic MDA-MB-231 tumors (Fig. 4i and j).

### Metformin improved the vascular functionality of metastatic breast cancers

**Fig. 5:**
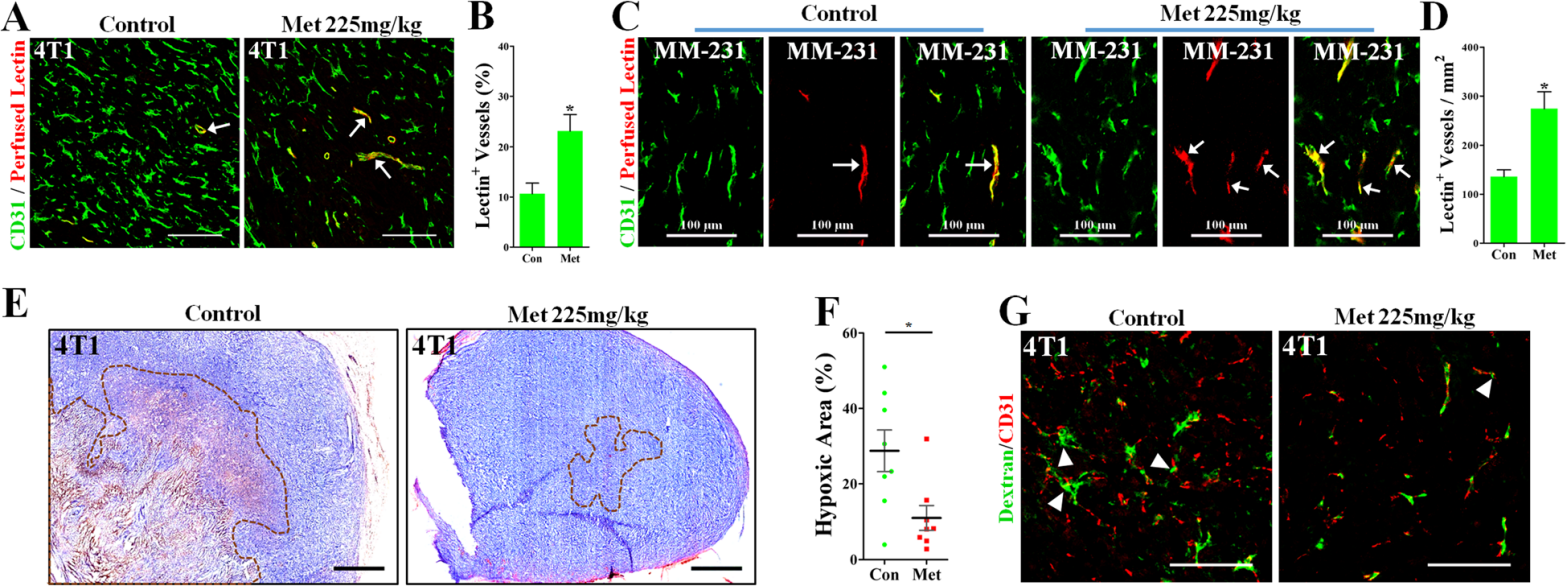
Effects of metformin administration on hypoxia and the vascular functionality. (**a**-**d**) Representative images showing more lectin-perfused CD31^+^ vessels in metformin-treated than untreated (A) 4T1 and (**c**) MDA-MB-231 cancers (n = 8). TRITC-conjugated lectin (Red) was intravenously injected into tail veins 15 min before the sacrifice. Tumor sections were further immunostained with anti-CD31 antibody (Green). White arrows indicate CD31^+^ vessels with lectin perfusion. Higher (B) percentage (of CD31^+^ vessels) and (**d**) density (per mm^2^) of Lectin^+^ CD31^+^ vessels in metformin-treated than untreated tumors. Bars: 100 μm. (**e**) Immunohistochemical staining for pimonidazole (PIMO; brown) and (**f**) quantification of PIMO^+^ hypoxic area in 4 T1 cancers from untreated or metformin-treated mice, revealing ameliorated tumor hypoxia by metformin (n = 8). Bars: 500 μm. (**g**) Representative images showing decreased vascular leakage in metformin-treated than untreated 4 T1 tumors. FITC-dextran (green) was injected through tail vein in advance. Tumor sections was counterstained with anti-CD31 antibody (Red). White triangles indicate the dextran leaking outside the vessel wall. Bars: 100 μm. Quantitative data are indicated as mean ± SEM. *p < 0.05

Structural maturity is closely associated with the vascular functional status [33]. We then focused on the effect of the administration of metformin on the perfusion status. A TRITC-conjugated Lectin, which can bind to the surface of ECs lining along the blood flow, was intravenously injected. Critically, both metformin-treated 4T1 and MDA-MB-231 tumors exhibited significantly higher percentage of Lectin^+^ vessels than those of the control (White arrows, Fig. 5a-d). This functional improvement was further validated by the fact that metformin greatly decreased tumor hypoxia (Fig. 5e and f), indicated by reduced positive areas of pimonidazole (PIMO). Furthermore, vessels in metformin-treated tumors were less leaky than in the control group (Fig. 5g). Collectively, these findings suggested that metformin improved the vessel functionality by increasing the vascular perfusion and decreasing the vascular leakiness, thus serving as a mediator for the normalization of breast cancer vessels.

### Metformin downregulated tumoral PDGF-B and reduced the vascular compression

**Fig. 6:**
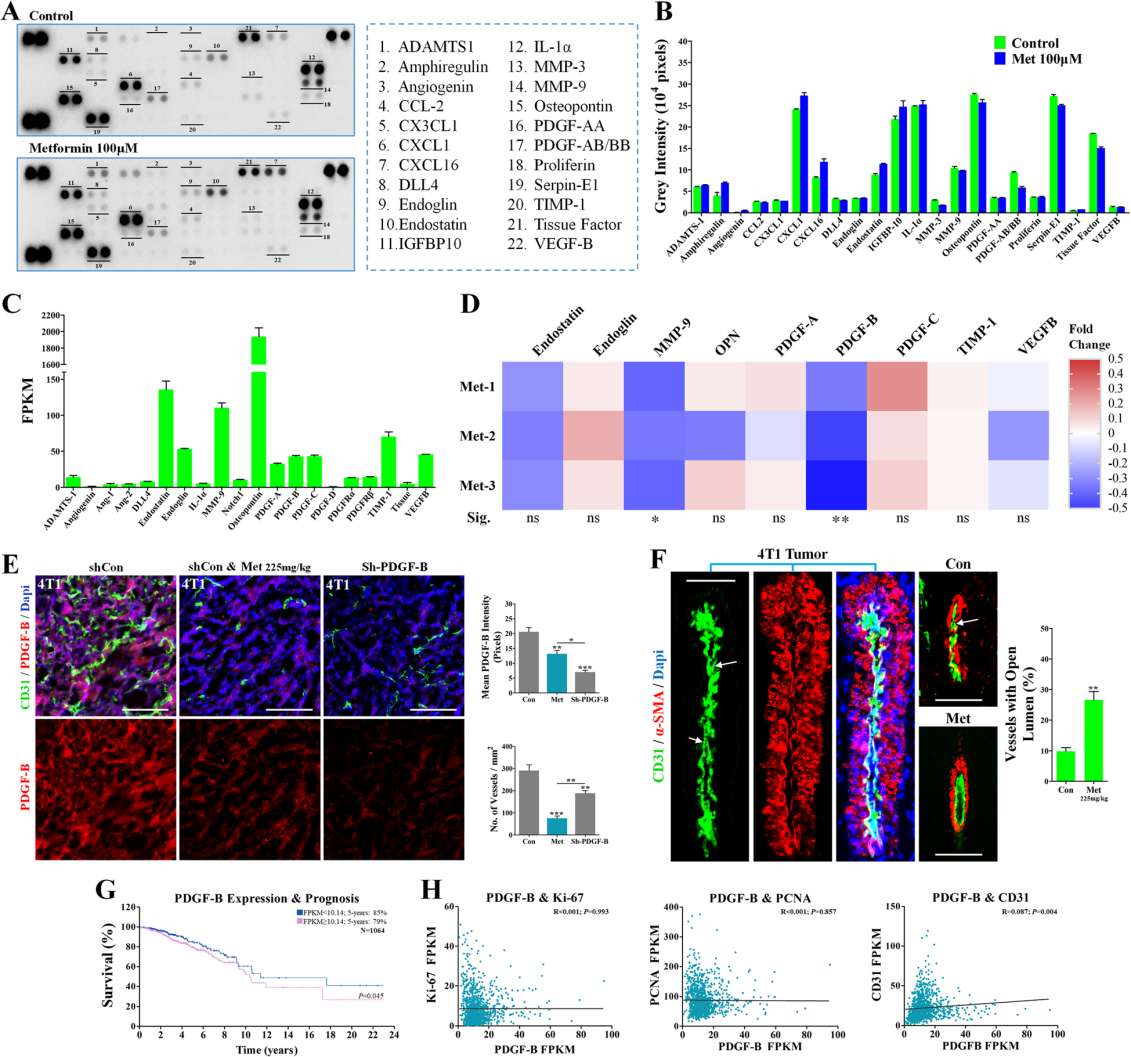
Downregulation of PDGF-B by the metformin treatment. (**a**) Immunoblotting for cancer cell-derived angiogenic factors using mouse angiogenesis arrays, and (**b**) quantification of grey intensity of each factor (independent of 3 experiments). Cancer cells were treated with metformin (100 μM) for 48 h. (**c**) Column chart for showing the transcriptional levels of angiogenesis-related factors. FPKM indicates Fragments Per Kilobase of transcript per Million fragments mapped. Met indicates metformin; Met1, Met2 and Met3 indicate different reports of data of independent tumors in metformin group. (**d**) Heatmap analysis of the effect of metformin on transcriptional levels of top 9 angiogenesis-related genes of 4T1 tumors (*n* = 3). The fold change ranges from − 0.5 to 0.5. “Sig.” indicates “Significance”. (**e**) Double immunostaining for CD31 and PDGF-B in 4T1 orthotopically implanted tumors (Left). shRNA-PDGF-B- and shRNA-Control-transfected 4T1 tumor-bearing mice received control or metformin treatment (225 mg/kg•day). Quantification of PDGF-B fluorescent intensity and microvessel density (Right; n = 8). (**f**) Immunostaining for CD31 and α-SMA in 4T1 cancers, revealing increased percentage of patent microarteries (n = 8). White arrows indicate the compressed lumen of arteries distributed in 4T1 cancer stroma. (**g**-**h**) Survival curve for analyzing the association of PDGF-B mRNA expression in primary breast tumor with survival probability of patients with breast cancer (**g**). PDGF-B mRNA levels was positively correlated with mRNA level of CD31, but not PCNA and Ki-67. Data was obtained from TCGA dataset. 1064 samples were included. FPKM: Fragments Per Kilobase of transcript per Million fragments mapped. Quantitative data are indicated as mean ± SEM. “ns” indicates not statistically significant; *p < 0.05; **p < 0.01; ***p < 0.001

Efforts were further devoted to the gaining of mechanistic insights into the metformin-induced vessel normalization. Excessive pro-angiogenic factors contributed to the abnormal angiogenesis [34, 35], resulting in an immature vasculature [36], which thus motivated us to investigate the underlying mechanism with a special focus on angiogenic factors. Results of the angiogenesis protein array showed that metformin treatment resulted in a great reduction of PDGF-B protein levels of 4T1 cancer cells in vitro (Fig. 6a and b), while the protein levels of some other factors were slightly affected. To validate this initial screening result, RNA sequencing was performed to detect the change of those affected factors in vivo. Expression levels of endoglin, endostatin, MMP-9 and osteopontin were higher than 50 FPKM (Fig. 6c), but not greatly affected. Among PDGFs, the expression of PDGF-B was the most significantly decreased (Fig. 6d), indicating the initial high PDGF-B expression in 4T1 cells. Consistently, metformin treatment resulted in a great reduction in the PDGF-B signal intensity of 4T1 tumors (Fig. 6e). These results were consistent with the finding of our previously published article that metformin reduced the PDGF-B signal intensity in the peri-necrotic regions of the 4 T1 tumor [37]. Overall, these data suggested PDGF-B downregulation was deeply involved in metformin’s vascular remodeling effects.

PDGF-B has been demonstrated to increase the interstitial fluid pressure (IFP), which is assumed to compress the intratumoral vessels [38], thus affecting the vascular functionality. In 4T1 tumors of the control group, those major arteries with multiple layers and VSMCs coverage were found to be severely compressed (Fig. 6f), with only very few arteries were patent with open lumen. Compared to the control group, the percentage of patent vessels was significantly elevated in 4T1 tumors of the metformin group (Fig. 6f). PDGF-B signaling has been reported to regulate the interstitial fluid pressure accompanied with the hyper-activated Hippo signaling. As was expected, the expression level of YAP was decreased by metformin in 4 T1 tumors (Additional file 1: Figure S1C). These evidences suggested that the down-regulation of PDGF-B by metformin might be contributed to reduce the compression of cancer cells to vessels.

### High PDGF-B expression was associated with poor prognosis and high CD31 expression

To determine if PDGF-B expression is associated with prognosis in female patients with breast cancers, the published TCGA dataset containing both gene expression and survival data was employed. 1064 patients were divided into high (≥10.14 FPKM) and low (< 10.14 FPKM) PDGF-B expression groups. High PDGF-B mRNA expression levels were associated with a significantly decreased 5-year survival rate (Fig. 6g). Further analysis for the relationship between mRNA levels of PDGF-B, PCNA, Ki-67 and CD31 was performed to investigate what contributed to the poor prognosis. Both PCNA and Ki-67 mRNA levels were not correlated with PDGF-B mRNA levels (Fig. 6h). Interestingly, increased CD31 mRNA levels were associated with significantly increased PDGF-B mRNA levels (Fig. 6h). These evidences suggested the contribution of PDGF-B to poor prognosis was associated with the vascular mechanism, but not the direct proliferative promotion of breast cancer cells.

### PDGF-B knockdown in breast cancers with high PDGF-B expression improved vascular maturity and function

**Fig. 7:**
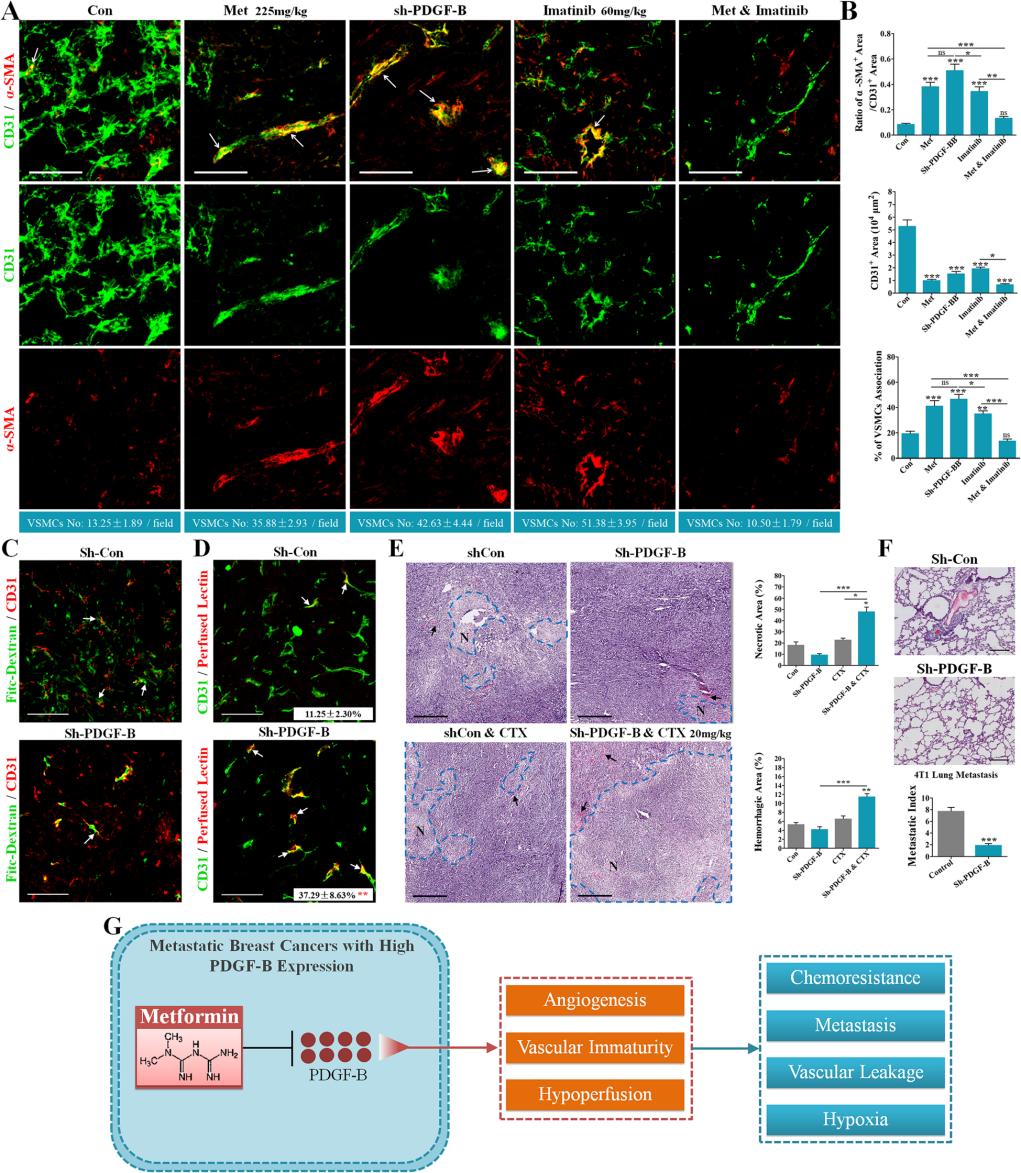
Effects of PDGF-B knockdown on metastasis, chemosensitization and vascular maturity and functionality. (**a**) Double staining for CD31 (green) and α-SMA (red) of 4T1 tumor sections. shRNA-PDGF-B- and shRNA-Control-transfected 4 T1 tumor-bearing mice received control, metformin (225 mg/kg), imatinib (60 mg/kg) for the combined treatment. Fluorescent signaling of series thin-layer scanning were reconstructed for 3D observation of VSMCs on vessels. VSMCs No. is at the bottom of the panel and indicated as mean ± SEM (n = 8). (**b**) Quantification of ratio of α-SMA^+^ area/CD31^+^ area (upper), CD31^+^ area per μm^2^ (middle) and percentage of α-SMA^+^ VSMCs associated with vessels (bottom) in 4 T1 cancers (n = 8). (**c**) Representative images showing decreased vascular leakage in shRNA-PDGF-B- than shRNA-Con-transfected 4T1 tumors. Fitc-dextran (green) was injected through tail vein 10 mins before sacrifice. Tumor sections was counterstained with anti-CD31 antibody (Red). White arrows indicate the dextran leaking outside the vessel wall. Bars: 100 μm. (**d**) Representative images showing more lectin-perfused CD31^+^ vessels in shRNA-PDGF-B- than shRNA-Con-transfected 4T1 tumors (n = 8). Red: perfused lectin; Green: CD31^+^ vessels. White arrows indicate CD31^+^ vessels with lectin perfusion. Percentage of Lectin^+^/CD31^+^ vessels (of CD31^+^ vessels) is indicated as mean ± SEM (at the bottom of the panel). (**e**) H&E staining of sections of 4T1 cancers (Left) and quantification of necrotic and hemorrhagic areas (Right; n = 8). shRNA-PDGF-B or shRNA-Con-transfected 4T1 tumor-bearing mice received low dose CTX (20 mg/kg•day). “N” indicates “necrosis”; black arrows indicate tumor hemorrhage. (**f**) Decreased primary tumor lung metastasis in mice bearing shRNA-PDGF-B 4T1 cancer cells than that in mice bearing shRNA-Con 4T1 cells. (Upper) H&E staining for 4T1 tumor sections; (Lower) quantification of 4T1 metastatic index (metastatic nodules per gram of primary tumor; n = 8). Red asterisk indicates lung metastasis nodule. Magnification: 200Х. (**g**) Schematic diagram of metformin-induced vascular remodeling in metastatic breast cancers. Metastatic breast cancers are angiogenic with hypoperfusion and vascular immaturity, which contribute to vascular leakage, chemoresistance, hypoxia and distant metastasis. By decreasing PDGF-B of those metastatic breast cancers, metformin inhibits angiogenesis and improved the vascular maturity and functionality, therefore improving the chemosensitivity and reducing the distant metastasis

To further validate if PDGF-B downregulation mediates the vessel normalization in cancers with PDGF-B expression, 4T1 cells were transfected by lentiviral shRNA-PDGF-B. As shown in Fig. 6e, PDGF-B knockdown greatly decreased the mean fluorescent PDGF-B intensity from 20.7 to 6.2 pixels in the sections of implanted tumors (Fig. 6e), indicating a successful knockdown of PDGF-B. Furthermore, PDGF-B knockdown markedly reduced the MVD and CD31^+^ areas of 4T1 cancers compared with the control group (Fig. 7a and b), suggesting the angiogenic effect of tumoral PDGF-B level. Similar with metformin, PDGF-B knockdown significantly increased percentages of α-SMA^+^ vessels and VSMCs, demonstrating the improved vascular maturity (Fig. 7a and b). In addition, vessels in shRNA-PDGF-B 4 T1 tumors were less leaky than those in shRNA-Con tumors (Fig. 7c), and there were more Lectin-perfused vessels in shRNA-PDGF-B 4 T1 tumors (Fig. 7d).

### PDGF-Rβ blockade abrogated metformin’s vascular remodeling effects

PDGFR-β is the receptor of PDGF-B and has been extensively studied for its critical role in vascular remodeling. Therefore, we then focused on exploring whether PDGF-Rβ was involved in metformin’s vascular remodeling effects. Imatinib, a drug that can specifically block PDGFR-β signaling, was used in this study. Similar to sh-PDGF-B and metformin, PDGFR-β blockade by imatinib led to decreased CD31^+^ areas and an increased vascular coverage of VSMCs (Fig. 7a and b). Surprisingly, imatinib apparently abrogated the metformin-induced increase of the VSMCs coverage on vessels (Fig. 7a and d), indicating the involvement of PDGFR-β signaling in metformin’s vascular remodeling effects. Notably, the combination of imatinib and metformin decreased the percentage of VSMCs associated with vessels to a comparable level of the shRNA control group (Figure7B). These evidences suggested that PDGFR-β is indispensable for the subsequent vascular remodeling when PDGF-B was downregulated.

### PDGF-B knockdown improved the chemosensitization of CTX and reduced lung metastasis

As PDGF-B knockdown induces the vessel normalization, we then investigated whether PDGF-B knockdown improves the chemosensitization and limits the primary lung metastasis of breast tumors. As shown in Fig. 7e, PDGF-B knockdown induced a slight reduction in 4T1 cancer necrosis without affecting hemorrhage. When treated with CTX, shRNA-PDGF-B 4T1 cancers exhibited a significant increase in both tumor necrosis and hemorrhage compared with those in the shRNA control group (Fig. 7e). Since increased blood perfusion can bring more drugs into the tumor tissue, these findings thus suggest that the inhibition of tumoral PDGF-B enhances chemosensitization by increasing the drug delivery owing to the improved vascular function. In further characterization of the primary lung metastasis (Fig. 7f), the metastatic index of the shRNA PDGF-B group was significantly reduced, compared to the shRNA control group.

## Discussion

Bevacizumab, an AAD approved at an early time, did not significantly improve the overall survival of patients with metastatic breast cancers [9, 39]. Bevacizumab was initially designed to neutralize the VEGF, thus inhibiting VEGFR-2-mediated angiogenesis and tumor growth. However, as previously reported, metastatic breast cancer cells had high expressions of several other pro-angiogenic factors in addition to VEGF [40], such as FGF-2, Ang-2 and PDGF-B [37]. Thus, in theory, AADs designed for targeting a single factor may be not enough [41], which offers an explanation for AAD’s ineffectiveness in treating cancers derived from some organ systems. Our laboratory previously found that metformin inhibited the expression of VEGF, Ang-2 and FGF-2 in a metastatic breast cancer model [37]. Besides, PDGF-B was screened out by transcriptome sequencing, and angiogenesis was greatly inhibited by PDGF-B knockdown. This result was validated by the clinical data that the PDGF-B expression level was positively associated with the CD31 expression level. Due to these findings, metformin should be considered as a reagent with a broad range of targeted factors possibly more available than the conventional AADs.

Another disadvantage of traditional AADs is the excessive pruning of the tumor vasculature [20], thus leading to the hypoxia-mediated tumor cell dissemination [42, 43]. Compared to AADs, metformin has more angiogenic targets, such as VEGF, PDGF-B and FGF-2. Therefore, metformin is assumed to prune the breast cancer vasculature more excessively. However, to date, it has not been reported that metformin aggravated tumor hypoxia [44]. Consistently, metformin ameliorated the hypoxia of two metastatic breast cancers, while MVD was greatly reduced. These evidences suggest there exists a mechanism independent of affecting the vasculature, which is accountable for the ameliorated hypoxia. Metformin is an AMPK activator that induces energetic stress [45]. In this condition, tumor cells were metabolically reprogrammed to consume less oxygen, thus counteracting or reversing the vascular pruning-induced hypoxia [44].

Currently, the issue of tumoral PDGF-B’s effect on the vascular maturity has become controversial [46]. Platelet-derived growth factor B (PDGF-B), a member of the PDGFs family [46], binds to its receptors, such as PDGF receptor β (PDGFR-β), to induce the cell survival, proliferation and migration [47]. It is now widely accepted that the endothelial PDGF-B regulates the recruitment of PCs [47]. Thus, the downregulation of PDGF-B should reduce the vascular maturity. However, PDGF-B was highly expressed by some tumors [48, 49], which were inversely characterized by an excessively angiogenic and immature vasculature. Further blockade of PDGF-B/PDGFR-β significantly increased the vascular maturity of tumors with high PDGF-B expression [49], but reduced that of tumors with low PDGF-B expression. These evidences indicated that high and low expression of PDGF-B in tumors might have opposite effects on vascular maturity. To date, this mechanism has not been reported in studies on metastatic breast cancers. Herein, it was found that high PDGF-B expression was detected in the metastatic breast cancer model. By reducing tumoral PDGF-B, the metformin treatment resulted in the suppression of angiogenesis and a more mature vasculature of metastatic breast cancers, thus limiting the distant metastasis and improving chemosensitization. These evidences are further supported by the poor prognosis of patients with breast cancers of high PDGF-B expression. These data indicate that the downregulation of PDGF-B in tumors with high expression of PDGF-B inhibits angiogenesis and improves the vascular functionality and maturity.

As early as in 1970’s, biguanides were reported to potentiate the antitumor effects of CTX and other chemo-drugs in vivo [21]. As shown in a recent clinical trial [50], diabetic patients receiving metformin had a greater response rate to chemo-drug than non-diabetic patients. Despite the increasing efforts that have been made [22], it was still unclear whether or how metformin sensitizes cancer cells to chemo-drugs. Recently, metformin has been demonstrated to directly enhance the toxicity of chemo-drugs [51–53]. However, concentrations used in those studies were higher than the blood concentration of patients. In the current paper, metformin was not found to significantly inhibit the proliferation and migration of 4 T1 cancer cells at the blood concentrations in vitro, indicating an indirect chemosensitization mechanism. Furthermore, our results suggest metformin-mediated chemosensitization resulted from the enhancement of drug delivery rather than the direct enhancement of the toxicity of chemo-drug.

Interestingly, metformin pretreatment results in a response of metastatic breast cancer cells to CTX at a lower dose, which was further supported by the increased CPT nucleus adduct^+^ cells. Critically, those CTX-induced apoptotic cells were located to the regions adjacent to vessels. Thus, metformin’s chemosensitization effect may be due to the increased delivery of chemo-drugs to the deep tumor. Vessel normalization activity increases the tumor oxygen and reduces the interstitial fluid pressure [36, 54], thus enhancing the generation of oxygen radicals and promoting egress of cytotoxic agents to the perivascular region in tumors. Consistent with the results from other laboratories [44], metformin treatment increased tumor oxygenation, reduced tumor hypoxia and improved radiotherapy response. Furthermore, metformin-induced chemosensitization may be contributed by the reduction in tumoral PDGF-B, which mediates resistance by PDGFR-β signaling and increasing IFP [55]. It has also been reported that metformin’s chemosensitization effect was contributed by increased uptake of chemotherapeutic by tumor cells [56].

Given that metformin has long been used for the treatment of type 2 diabetes mellitus, the results of this research is remarkable in terms of drug re-positioning (DR) [57]. DR is a screening for anti-cancer therapeutic effects of conventionally administered medications for non-malignant disorders, which has attracted a great deal of attention as the safety and frequency of side effects of these medicines have been already proven. For a typical instance, ticlopidine (purinergic receptor P2Y12 inhibitor), which is an anti-coagulant drug to prevent the transient ischemic attack (TIA) and stroke, and has been shown to be effective for low-grade glioma and high-grade astrocytoma. This P2Y12 inhibitor increases the intracellular cAMP level and promotes the autophagy flux [58]. Notably, tricyclic antidepressants such as imipramine promote autophagy in glioma cells synergistically with this drug by further elevating the intracellular cAMP concentration [59].

Metformin activates AMPK signal pathway, which not only decreases insulin resistance in type 2 diabetes mellitus but also blocks AMPK-mediated mTOR activation even in cancer stem cells (CSCs) [60]. mTOR signal is regulated by amino-acid transporters [61], characterized by the L-type amino acid transporter 1 (LAT1; SLC7A5) and the glutamine/amino acid transporter (ASCT2; SLC1A5), which explains why the AMPK-mTOR axis functions as a sensor of the dynamic change in the nutrient/growth factor microenvironment. Particularly, the leucine uptake via LAT1 activates the mTOR signal pathway leading to poor prognosis. Because EpCAM is a functional CSC marker that forms a complex with amino-acid transporters such as LAT1 [62], it is reasonable that the LAT1 expression level would be positively correlated with poor prognosis. Therefore, the LKB1-AMPK-mTOR axis is orchestrated by the amino-acid concentration in the tumor microenvironment, and this axis promotes the metabolic reprogramming of cancer cells in response to the microenvironment [58, 63].

More clinical and pre-clinical evidences should be provided to validate the vascular mechanism, and if metformin targets a broad range of angiogenesis-related factors. As metformin is a drug widely prescribed for metabolic disorder, further efforts should also be devoted to investigating the involvement of the metabolic mechanism and its contribution to ameliorated hypoxia.

## Conclusions

Our current work provided the pre-clinical evidences for metformin’s effect in remodeling the abnormal breast cancer vasculature. Herein, the antidiabetic agent metformin inhibited the progression of metastatic breast cancers, and induced chemosensitization by a vascular mechanism. By decreasing tumoral PDGF-B, metformin inhibited the excessive angiogenesis and improved the vascular maturity and functionality. The normalized vasculature was with more mural cells coverage and better basement membrane [30], thus limiting distant metastasis. As the structure determines the function, improved vascular maturity led to an increase in the blood perfusion of tumors, thus allowing more chemo-drugs or therapeutic particles to be delivered into the tumors [64]. On the basis of that, the elucidated vascular mechanism of metformin is of great significance and value for the clinical treatment of metastatic breast cancers.

## Additional file

Additional file 1: **Figure S1.** Effects of metformin on expression level of YAP and discrepancy in hypoxia between hypo-vascular and hyper-vascular regions. (DOCX 1571 kb)

## Abbreviations

3D: 3-dimension
4-OH-CTX: 4-hydroxy-cyclophosphamide
AADs: Anti-angiogenic drugs
ATCC: American Type Culture Collection
CD31: Alternative name of PECAM-1, platelet endothelial adhesion molecule
CPT: Cisplatin
CPT-1: Carnitine acyltransferase 1
CTX: Cyclophosphamide
DAB: 3,3′-diaminobenzidine
DAPI: 4′,6-diamidino-2-phenylindole
DMEM: Dulbecco modified eagle medium
DR: Drug re-positioning
ECL: Enhanced chemiluminescence
ECs: Endothelial cells
EpCAM: Epithelial cell adhesion molecule
FBS: Fetal bovine serum
FGF-2: Fibroblast growth factor-2
GFP: Green fluorescent protein
H&E staining: Hematoxylin and eosin staining
HRP: Horseradish peroxidase
ICLAC: International Cell Line Authentication Committee
IF: Immunofluorescence
IFP: Interstitial fluid pressure
MM-231: MDA-MB-231
MMP: Metalloproteinase
MVD: Microvessel density
OCT: Optimal cutting temperature compound
PARP: Poly-ADP-ribose polymerase
PBS: Phosphate-buffered solution
PCNA: Proliferating cell nuclear antigen
PCR: Polymerase chain reaction
PDGF: Platelet-derived growth factor
PDGFR-β: Platelet-derived growth factor receptor-β
PFK: Phosphofructokinase
PI: Propidium iodide
PIMO: Pimonidazole
shRNA: Small hairpin RNA
STR: Short Tandem Repeat
TCGA: The cancer genome atlas
TRITC: Tetramethyl rhodamin isothiocyanate
VE-cadherin: Vascular endothelial-cadherin
VEGF: Vascular endothelial growth factor
VSMCs: Vascular smooth muscle cells
α-SMA: Smooth muscle actin α

## References

[1] Kerbel RS (2008). Tumor angiogenesis. N Engl J Med.

[2] FOLKMAN J (2002). Role of angiogenesis in tumor growth and metastasis. Seminars in Oncology.

[3] Ebos JML, Kerbel RS (2011). Antiangiogenic therapy: impact on invasion, disease progression, and metastasis. Nat Rev Clin Oncol.

[4] Goel S, Duda DG, Xu L, Munn LL, Boucher Y, Fukumura D, Jain RK (2011). Normalization of the vasculature for treatment of cancer and other diseases. Physiol Rev.

[5] Ouarne M, Bouvard C, Boneva G, Mallet C, Ribeiro J, Desroches-Castan A, Soleilhac E, Tillet E, Peyruchaud O, Bailly S (2018). BMP9, but not BMP10, acts as a quiescence factor on tumor growth, vessel normalization and metastasis in a mouse model of breast cancer. J Exp Clin Cancer Res.

[6] Jain RK (2014). Antiangiogenesis strategies revisited: from starving tumors to alleviating hypoxia. Cancer Cell.

[7] Willett CG, Boucher Y, Di Tomaso E, Duda DG, Munn LL, Tong RT, Chung DC, Sahani DV, Kalva SP, Kozin SV (2004). Direct evidence that the VEGF-specific antibody bevacizumab has antivascular effects in human rectal cancer. Nat Med.

[8] Bear HD, Tang G, Rastogi P, Geyer CE, Robidoux A, Atkins JN, Baez-Diaz L, Brufsky AM, Mehta RS, Fehrenbacher L (2012). Bevacizumab added to neoadjuvant chemotherapy for breast cancer. N Engl J Med.

[9] Miller K, Wang M, Gralow J, Dickler M, Cobleigh M, Perez EA, Shenkier T, Cella D, Davidson NE (2007). Paclitaxel plus bevacizumab versus paclitaxel alone for metastatic breast cancer. N Engl J Med.

[10] Tolaney SM, Boucher Y, Duda DG, Martin JD, Seano G, Ancukiewicz M, Barry WT, Goel S, Lahdenrata J, Isakoff SJ (2015). Role of vascular density and normalization in response to neoadjuvant bevacizumab and chemotherapy in breast cancer patients. Proc Natl Acad Sci.

[11] Rapisarda A, Melillo G (2012). Overcoming disappointing results with antiangiogenic therapy by targeting hypoxia. Nat Rev Clin Oncol.

[12] Kisfalvi K, Eibl G, Sinnett-Smith J, Rozengurt E (2009). Metformin disrupts crosstalk between G protein-coupled receptor and insulin receptor signaling systems and inhibits pancreatic cancer growth. Cancer Res.

[13] Gonzalez-Angulo AM, Meric-Bernstam F (2010). Metformin: a therapeutic opportunity in breast cancer. Clin Cancer Res.

[14] Daugan M, Dufaÿ Wojcicki A, d’Hayer B, Boudy V (2016). Metformin: An anti-diabetic drug to fight cancer. Pharmacol Res.

[15] Martin-Castillo B, Vazquez-Martin A, Oliveras-Ferraros C, Menendez JA (2010). Metformin and cancer: doses, mechanisms and the dandelion and hormetic phenomena. Cell Cycle.

[16] Pan Q, Yang GL, Yang JH, Lin SL, Liu N, Liu SS, Liu MY, Zhang LH, Huang YR, Shen RL (2015). Metformin can block precancerous progression to invasive tumors of bladder through inhibiting STAT3-mediated signaling pathways. J Exp Clin Cancer Res.

[17] Qian W, Li J, Chen K, Jiang Z, Cheng L, Zhou C, Yan B, Cao J, Ma Q, Duan W (2018). Metformin suppresses tumor angiogenesis and enhances the chemosensitivity of gemcitabine in a genetically engineered mouse model of pancreatic cancer. Life Sci.

[18] Garrido Maritza P., Vera Carolina, Vega Margarita, Quest Andrew F.G., Romero Carmen (2018). Metformin prevents nerve growth factor-dependent proliferative and proangiogenic effects in epithelial ovarian cancer cells and endothelial cells. Therapeutic Advances in Medical Oncology.

[19] Han J, Li Y, Liu X, Zhou T, Sun H, Edwards P, Gao H, Yu FS, Qiao X (2018). Metformin suppresses retinal angiogenesis and inflammation in vitro and in vivo. PLoS One.

[20] Carmeliet P, Jain RK (2011). Principles and mechanisms of vessel normalization for cancer and other angiogenic diseases. Nat Rev Drug Discov.

[21] Dilman V, Anisimov V (1979). Potentiation of antitumor effect of cyclophosphamide and hydrazine sulfate by treatment with the antidiabetic agent, 1-phenylethylbiguanide (phenformin). Cancer Lett.

[22] Lin YC, Wu MH, Wei TT, Lin YC, Huang WC, Huang LY, Lin YT, Chen CC (2014). Metformin sensitizes anticancer effect of dasatinib in head and neck squamous cell carcinoma cells through AMPK-dependent ER stress. Oncotarget.

[23] Peter G, Wagner T, Hohorst HJ (1976). Studies on 4-hydroperoxycyclophosphamide (NSC-181815): a simple preparation method and its application for the synthesis of a new class of "activated" sulfur-containing cyclophosphamide (NSC-26271) derivatives. Cancer Treat Rep.

[24] Wagner T, Peter G, Voelcker G, Hohorst HJ (1977). Characterization and quantitative estimation of activated cyclophosphamide in blood and urine. Cancer Res.

[25] Fischer C, Jonckx B, Mazzone M, Zacchigna S, Loges S, Pattarini L, Chorianopoulos E, Liesenborghs L, Koch M, De Mol M (2007). Anti-PlGF inhibits growth of VEGF(R)-inhibitor-resistant tumors without affecting healthy vessels. Cell.

[26] Jain RK, Booth MF (2003). What brings pericytes to tumor vessels?. J Clin Investig.

[27] Raza A, Franklin MJ, Dudek AZ (2010). Pericytes and vessel maturation during tumor angiogenesis and metastasis. Am J Hematol.

[28] Reagan-Shaw S, Nihal M, Ahmad N (2008). Dose translation from animal to human studies revisited. FASEB J.

[29] Hicklin Daniel J., Ellis Lee M. (2005). Role of the Vascular Endothelial Growth Factor Pathway in Tumor Growth and Angiogenesis. Journal of Clinical Oncology.

[30] Maes H, Kuchnio A, Peric A, Moens S, Nys K, De Bock K, Quaegebeur A, Schoors S, Georgiadou M, Wouters J (2014). Tumor vessel normalization by chloroquine independent of autophagy. Cancer Cell.

[31] Chauhan VP, Stylianopoulos T, Martin JD, Popovic Z, Chen O, Kamoun WS, Bawendi MG, Fukumura D, Jain RK (2012). Normalization of tumour blood vessels improves the delivery of nanomedicines in a size-dependent manner. Nat Nanotechnol.

[32] Huang Y, Yuan J, Righi E, Kamoun WS, Ancukiewicz M, Nezivar J, Santosuosso M, Martin JD, Martin MR, Vianello F (2012). Vascular normalizing doses of antiangiogenic treatment reprogram the immunosuppressive tumor microenvironment and enhance immunotherapy. Proc Natl Acad Sci.

[33] Carmeliet P, Jain RK (2011). Molecular mechanisms and clinical applications of angiogenesis. Nature.

[34] Carmeliet P (2005). Angiogenesis in life, disease and medicine. Nature.

[35] Jayson GC, Kerbel R, Ellis LM, Harris AL. Antiangiogenic therapy in oncology: current status and future directions. Lancet. 2016. 10.1016/S0140-6736(15)01088-0 26853587

[36] Tong RT, Boucher Y, Kozin SV, Winkler F, Hicklin DJ, Jain RK (2004). Vascular normalization by vascular endothelial growth factor receptor 2 blockade induces a pressure gradient across the vasculature and improves drug penetration in tumors. Cancer Res.

[37] Wang JC, Li GY, Li PP, Sun X, Li WM, Li Y, Lu SY, Liu PJ (2017). Suppression of hypoxia-induced excessive angiogenesis by metformin via elevating tumor blood perfusion. Oncotarget.

[38] Chauhan Vikash P, Boucher Y, Ferrone Cristina R, Roberge S, Martin John D, Stylianopoulos T, Bardeesy N, DePinho Ronald A, Padera Timothy P, Munn Lance L, Jain Rakesh K (2014). Compression of pancreatic tumor blood vessels by Hyaluronan is caused by solid stress and not interstitial fluid pressure. Cancer Cell.

[39] Sledge GW (2015). Anti–vascular endothelial growth factor therapy in breast Cancer: game over?. J Clin Oncol.

[40] Wang J, Li G, Wang Y, Tang S, Sun X, Feng X, Li Y, Bao G, Li P, Mao X (2015). Suppression of tumor angiogenesis by metformin treatment via a mechanism linked to targeting of HER2/HIF-1alpha/VEGF secretion axis. Oncotarget.

[41] Mitsuhashi A, Goto H, Saijo A, Trung VT, Aono Y, Ogino H, Kuramoto T, Tabata S, Uehara H, Izumi K (2015). Fibrocyte-like cells mediate acquired resistance to anti-angiogenic therapy with bevacizumab. Nat Commun.

[42] Cassavaugh Jessica, Lounsbury Karen M. (2011). Hypoxia-mediated biological control. Journal of Cellular Biochemistry.

[43] Conley S. J., Gheordunescu E., Kakarala P., Newman B., Korkaya H., Heath A. N., Clouthier S. G., Wicha M. S. (2012). Antiangiogenic agents increase breast cancer stem cells via the generation of tumor hypoxia. Proceedings of the National Academy of Sciences.

[44] Zannella VE, Dal Pra A, Muaddi H, McKee TD, Stapleton S, Sykes J, Glicksman R, Chaib S, Zamiara P, Milosevic M (2013). Reprogramming metabolism with metformin improves tumor oxygenation and radiotherapy response. Clin Cancer Res.

[45] Liu X, Romero IL, Litchfield LM, Lengyel E, Locasale JW (2016). Metformin targets central carbon metabolism and reveals mitochondrial requirements in human cancers. Cell Metab.

[46] Cao Y (2013). Multifarious functions of PDGFs and PDGFRs in tumor growth and metastasis. Trends Mol Med.

[47] Kazlauskas A (2017). PDGFs and their receptors. Gene.

[48] Cao R, Björndahl MA, Religa P, Clasper S, Garvin S, Galter D, Meister B, Ikomi F, Tritsaris K, Dissing S (2004). PDGF-BB induces intratumoral lymphangiogenesis and promotes lymphatic metastasis. Cancer Cell.

[49] Hosaka K, Yang Y, Seki T, Nakamura M, Andersson P, Rouhi P, Yang X, Jensen L, Lim S, Feng N. Tumour PDGF-BB expression levels determine dual effects of anti-PDGF drugs on vascular remodelling and metastasis. Nat Commun. 2013;4. 10.1038/ncomms3129 23831851

[50] Jiralerspong S, Palla SL, Giordano SH, Meric-Bernstam F, Liedtke C, Barnett CM, Hsu L, Hung M-C, Hortobagyi GN, Gonzalez-Angulo AM (2009). Metformin and pathologic complete responses to neoadjuvant chemotherapy in diabetic patients with breast cancer. J Clin Oncol.

[51] Li L, Han R, Xiao H, Lin C, Wang Y, Liu H, Li K, Chen H, Sun F, Yang Z (2014). Metformin sensitizes EGFR-TKI–resistant human lung Cancer cells in vitro and in vivo through inhibition of IL-6 signaling and EMT reversal. Clin Cancer Res.

[52] Choi YW, Lim IK (2014). Sensitization of metformin-cytotoxicity by dichloroacetate via reprogramming glucose metabolism in cancer cells. Cancer Lett.

[53] Bai M, Yang L, Liao H, Liang X, Xie B, Xiong J, Tao X, Chen X, Cheng Y, Chen X (2018). Metformin sensitizes endometrial cancer cells to chemotherapy through IDH1-induced Nrf2 expression via an epigenetic mechanism. Oncogene.

[54] de Oliveira RL, Deschoemaeker S, Henze A-T, Debackere K, Finisguerra V, Takeda Y, Roncal C, Dettori D, Tack E, Jönsson Y (2012). Gene-targeting of Phd2 improves tumor response to chemotherapy and prevents side-toxicity. Cancer Cell.

[55] Pietras K, Rubin K, Sjöblom T, Buchdunger E, Sjöquist M, Heldin C-H, Östman A (2002). Inhibition of PDGF receptor signaling in tumor stroma enhances antitumor effect of chemotherapy. Cancer Res.

[56] Davies G, Lobanova L, Dawicki W, Groot G, Gordon JR, Bowen M, Harkness T, Arnason T (2017). Metformin inhibits the development, and promotes the resensitization, of treatment-resistant breast cancer. PLoS One.

[57] Yoshida GJ (2017). Therapeutic strategies of drug repositioning targeting autophagy to induce cancer cell death: from pathophysiology to treatment. J Hematol Oncol.

[58] Yoshida GJ (2015). Metabolic reprogramming: the emerging concept and associated therapeutic strategies. J Exp Clin Cancer Res.

[59] Shchors K, Massaras A, Hanahan D (2015). Dual targeting of the Autophagic regulatory circuitry in gliomas with repurposed drugs elicits cell-lethal autophagy and therapeutic benefit. Cancer Cell.

[60] Barbieri F, Thellung S, Ratto A, Carra E, Marini V, Fucile C, Bajetto A, Pattarozzi A, Wurth R, Gatti M (2015). In vitro and in vivo antiproliferative activity of metformin on stem-like cells isolated from spontaneous canine mammary carcinomas: translational implications for human tumors. BMC Cancer.

[61] Wullschleger S, Loewith R, Hall MN (2006). TOR signaling in growth and metabolism. Cell.

[62] Yoshida GJ, Saya H (2014). EpCAM expression in the prostate cancer makes the difference in the response to growth factors. Biochem Biophys Res Commun.

[63] Xu D, Hemler ME (2005). Metabolic activation-related CD147-CD98 complex. Mol Cell Proteomics.

[64] Jiang W, Huang Y, An Y, Kim BY (2015). Remodeling tumor vasculature to enhance delivery of intermediate-sized nanoparticles. ACS Nano.

