# Supplementary material for: Metformin inhibits metastatic breast cancer progression and improves chemosensitivity by inducing vessel normalization via PDGF-B downregulation
Source: J Exp Clin Cancer Res. 2019 Jun 4;38:235. doi: 10.1186/s13046-019-1211-2 (PMC6549289; doi:10.1186/s13046-019-1211-2)
Supplement: Supplementary file 1 — Figure S1. Effects of metformin on expression level of YAP and discrepancy in hypoxia between hypo-vascular and hyper-vascular regions. (DOCX 1571 kb) [file 13046_2019_1211_MOESM1_ESM.docx]

**
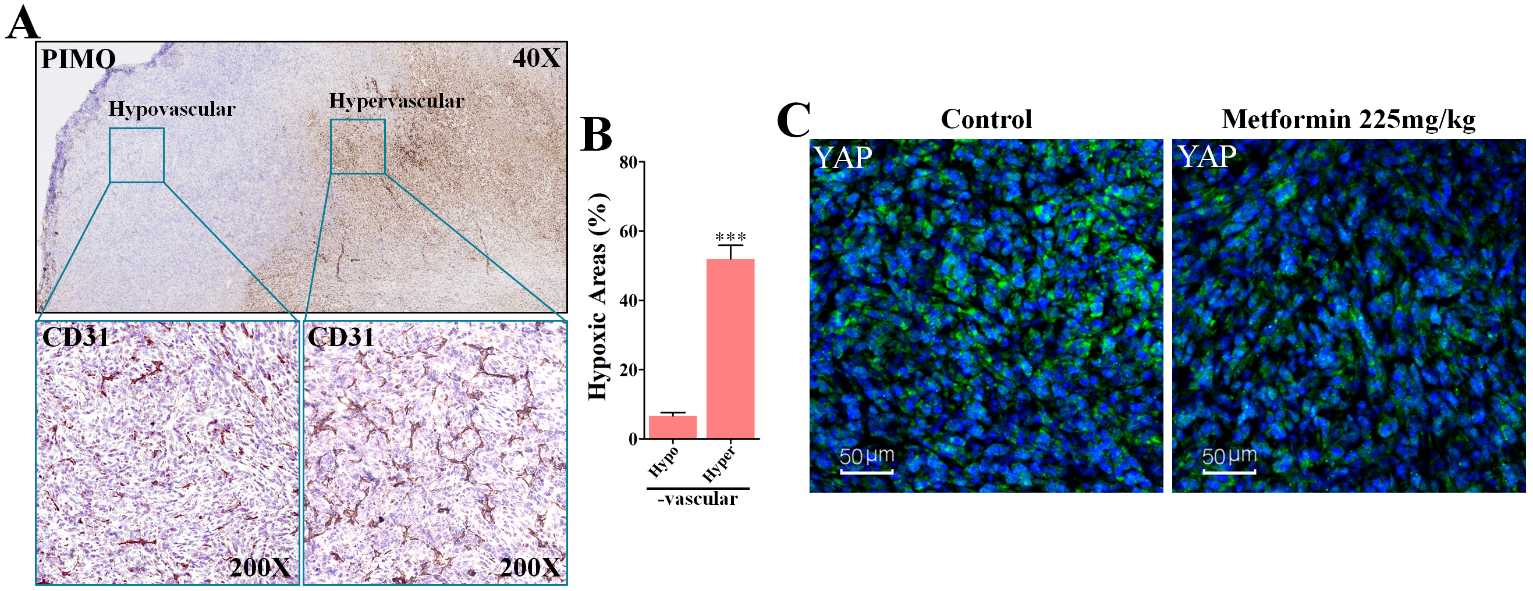
Supplementary Figure and Legend**

**Fig. S1**. **Effects of metformin on expression level of YAP and discrepancy in hypoxia between hypo-vascular and hyper-vascular regions.**

(A) Immunostaining for PIMO (the upper image, magnification: 40X) and CD31 (the lower images, magnification: 200X) in sections of 4T1 tumors from untreated mice. The lower-left and lower-right images indicate hypo-vascular and hyper-vascular regions, respectively. (B) Quantification of hypoxic areas (%) in both hypo- and hyper-vascular regions of 4T1 tumors (n=8). Quantitative data are indicated as mean ± SEM. ***p < 0.001. (C) Immunostaining for YAP in sections of 4T1 tumors from control-treated mice or 225 mg/kg•day metformin-treated mice. Metformin administration decreased the expression level of YAP in 4T1 tumors. Scale bar: 50μm.
